# Effect of Different Reduced Training Frequencies after 12 Weeks of Concurrent Resistance and Aerobic Training on Muscle Strength and Morphology

**DOI:** 10.3390/sports12070198

**Published:** 2024-07-22

**Authors:** Thomas Mpampoulis, Angeliki N. Stasinaki, Spyridon Methenitis, Nikolaos Zaras, Gregory C. Bogdanis, Gerasimos Terzis

**Affiliations:** 1 Sports Performance Laboratory, School of Physical Education & Sports Science, National and Kapodistrian University of Athens, Daphne, 17237 Athens, Greece; thompamp@phed.uoa.gr (T.M.); agstasin@phed.uoa.gr (A.N.S.); smetheni@phed.uoa.gr (S.M.); gbogdanis@phed.uoa.gr (G.C.B.); 2 Department of Physical Education and Sport Science, Democritus University of Thrace, 69100 Komotini, Greece; nzaras@phyed.duth.gr; 3 Human Performance Laboratory, Department of Life Sciences, School of Life and Health Sciences, University of Nicosia, Nicosia 2417, Cyprus

**Keywords:** detraining, muscle hypertrophy, cardiovascular endurance, high-intensity training

## Abstract

The aim of the study was to investigate the effect of two long-term reduced concurrent training modalities, in which participants performed one training session every either 7 or 14 days, after 12 weeks of systematic concurrent resistance and aerobic training, on lower extremities’ muscle strength, power, and morphology in young females. After the 12-week training period, participants were assigned into three groups and performed either one training session every 7 days (G7) or once every 14 days (G14), or detraining (GD), for 12 weeks, followed by 12 additional weeks of detraining. The following were measured before, after the systematic training period, after the end of the reduced training frequency period, and after the end of complete detraining: body composition, leg press 1-RM, countermovement jump, quadriceps cross-sectional area (CSA), vastus lateralis muscle architecture, and maximum aerobic power. Performance and muscle mass increased after the initial 12-week training period. Thereafter, leg press 1-RM, quadriceps CSA, and aerobic power remained unchanged in the G7 group, but decreased in G14 (−4.4 ± 3.5%; −5.9 ± 1.8%; −9.0 ± 7.8%, respectively, *p* < 0.05), maintaining 95.6 ± 3.5%, 94.1 ± 1.8%, and 91.0 ± 7.8% of the initial training adaptations, respectively. In conclusion, performing one training session every 2 weeks for 3 months may preserve 90 to 95% of the muscle mass/strength and aerobic power adaptations achieved with systematic concurrent training.

## 1. Introduction

Chronic exposure to combined resistance and aerobic training results in increased muscle strength and mass, bone density, and cardiovascular endurance, as well as improvements in metabolism and mental health [1]. These adaptations are essential for health and athletic performance in individuals independent of age, gender, and health status [2]. Indeed, it is well documented that even a low-volume, high-intensity combined resistance and aerobic training, in young females, two times per week, for several weeks, results in significant increases in muscle strength, mass, and aerobic capacity [3]. Similar results have been documented in older females after 12 weeks of combined resistance and aerobic training [4]. Thus, combined resistance and aerobic training is recommended by the American College of Sports Medicine [1], the American Heart Association [5], and the American Diabetes Association [6], and a large number of training enthusiasts follow this training paradigm. Specifically, according to the newest exercise/training guidelines, a total of 150 min moderate-intensity cardiorespiratory exercise training per week and resistance exercises for each of the major muscle groups 2 times per week are necessary to improve and maintain physical fitness and health in adults [1]. 

Unfortunately, despite the well-known benefits of regular, long-term training in health and physical fitness, it is very common that many trainees, after the initial period of their engagement with regular training, are forced to stop or decrease their training sessions per week. Indeed, several studies have reported that there is a dropout rate between 40% and 65% in individuals who started to participate in regular/systematic training programs after the initial 5 to 8 months [7,8]. Reduced training frequency or complete exercise cessation (detraining) results in the gradual loss of some or all of the acquired training adaptations [9]. The human body adapts to the reduced amount and intensity of physical activity by reducing muscle mass, strength/power, cardiovascular endurance, bone density, etc. [10], while the training-induced health benefits on cardiometabolic risk factors are soon abolished, especially if the training period was short-termed [11,12,13]. For example, at least in previously sedentary individuals, the training-induced beneficial changes in body composition, cardiovascular fitness, and lipidemic/glycemic blood profiles returned to pretraining values in just 2–4 weeks of detraining [11,12], while the observed increases in muscle strength and power, after 8 and 16 weeks of regular training, were significantly decreased after only 4 weeks of detraining [14,15]. Thus, considering the above, as well as the fact that the most important issue, in modern times, is not only to have significant improvements in body fitness and health status after a short-term engagement in systematic training programs but also to maintain the beneficial training-induced adaptations on the body composition, cardiorespiratory fitness, lipidemic/glycemic blood profiles, muscle strength/power, for as long as possible, it is of clinical and practical importance to investigate the minimum necessary frequency dose of training which could maintain the above adaptations during a period of forced or not decreased training frequency. The partial application of exercise during the detraining period might reverse the gradual loss of the acquired training adaptations [16], but data regarding this issue are very few, and mainly focused on athletes, or after short-term detraining periods, with their conclusions being very difficult to be generalized in non-athletic populations or to provide us a clear picture of the effect of long-term reduced training frequency. From the limited amount of currently available data on well-trained athletic populations, it seems that if the intensity and/or the volume of the training are kept high or at least at the same points as those during the end of the regular training period, muscular strength [16,17,18,19,20], cardiovascular fitness/aerobic performance [21,22], and quadriceps hypertrophy [23] could be maintained, even if the training frequency is reduced by 50–75%, i.e., from two training sessions to one or from three training sessions to two. However, if this is all true in a non-athletic population, who exercises in gym facilities, performs workouts addressed to the general population, and must overcome their daily needs and obligations, remains to be investigated. Furthermore, even if it is well known that training significantly increases bone mineral density, while the lack of physical activity leads to its reduction, until now, it remains unknown if the training-induced adaptations on bone mineral density may or may not be reserved during a long-term period of forced decreased training frequency. 

Considering the above, it seems that during a short-term period of reduced training frequency, the training-induced adaptations may, at least to some extent, be maintained. However, these conclusions are based on studies investigating the effect of the reduced training frequency of very specific training programs aiming to increase or to maintain either muscle strength/power, hypertrophy, aerobic capacity, etc. Thus, until now, it is largely unknown whether long-term reduced training frequency of concurrent resistance and aerobic training may preserve the concurrent training-induced adaptations in both neuromuscular and aerobic performances due to the concurrent training effect [24]. Moreover, until now, it is unknown how the different reduced training frequencies could affect the magnitude of the training-induced adaptation losses during a period of complete cessation of exercise. Thus, the aim of the present study was to investigate the effect of two long-term reduced concurrent training modalities, in which participants performed one training session every either 7 or 14 days, after 12 weeks of systematic concurrent resistance and aerobic training, on lower extremities’ muscle strength, power, and morphology in young females. It was hypothesized that training every 7 days during the reduced training period would preserve most of the muscle mass and strength while training every 14 days would result in a greater loss of muscle strength and mass. Furthermore, the present study also aimed to investigate how the above training modalities may affect the adaptation losses during a period of complete cessation of exercise, following a period of reduced training frequency.

## 2. Materials and Methods

### 2.1. Experimental Approach 

**Figure 1:**
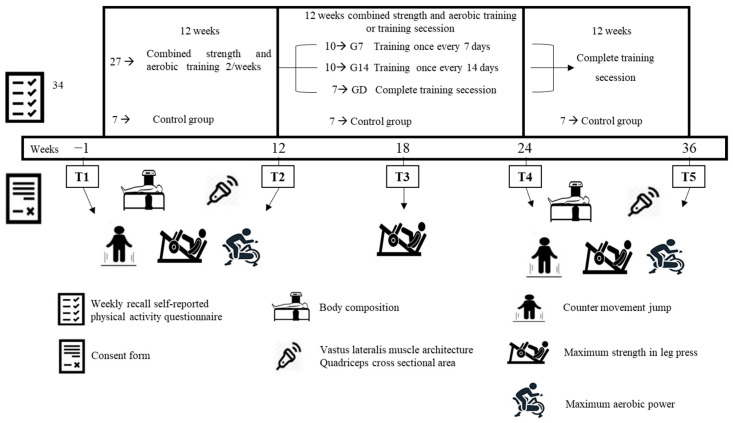
The experimental design of the present study.

Participants were recruited via advertisements in university student societies. The responders visited the laboratory, where they completed a weekly recall self-reported physical activity questionnaire to verify if the inclusion criteria of no systematic training were met. Thirty-four female students who met the inclusion criteria (only healthy females, aged between 18 and 26, with no systematic experience in resistance or aerobic training for at least 2 years) visited the laboratory for a second time three days later. They were fully informed about the study procedure and protocol and signed an informed consent form. On the same day, body height and mass were measured, and they performed a familiarization session for all the performance evaluations. Then, during their third, fourth, and fifth visits, all the evaluations were performed. Thereafter, the participants were randomly allocated into two groups (based on their body composition muscle strength/power, and aerobic capacity (no differences between groups; *p* > 0.05)), (A) training group: twenty-seven participants performed a 12-week combined resistance and aerobic training (two training session per week with 72 h intervals between them) and control group (GC; seven participants) who did not perform any kind of training. After this period, the participants of the training group (n = 27) were divided into 3 sub-groups and continued for another 12 weeks the same training protocol as above, as follows: group G7, who trained once per week (n = 10); group G14, who trained once every 14 days (n = 10); and detraining group (GD) with the complete cessation of training (n = 7, Figure 1). The allocation of the participants into the three sub-groups was performed based on the post-training values of the above parameters (no difference between groups; *p* > 0.05). After this, all the participants followed a 12-week period of complete training cessation. The evaluations of body composition, vastus lateralis muscle architecture, quadriceps cross-sectional area (CSA), leg press maximum strength (1-RM), countermovement jump (CMJ), and cycling maximal aerobic power were performed, with the same order and at the same conditions, one week before (T1) and after (T2) systematic training period, as well as one week after the end of the reduced training (T4) and detraining (T5) periods. Specifically, during the morning hours of the first testing day, body composition, quadriceps CSA, and vastus lateralis muscle architecture were evaluated. The following day, performance in CMJ and leg press 1-RM were measured. Two days later, maximal aerobic power was evaluated. An additional evaluation of leg press 1-RM was performed 6 weeks after the initiation of the reduced training period (T3) to identify a potential difference in the rate of a possible performance decline between the groups (Figure 1).

### 2.2. Participants

An a priori power analysis, based on the design of the present study and data from the pilot study, revealed that a total of 30 individuals have to participate and complete the study (for the two-way repeated analysis of variance) for an actual power of 0.900 for the results of the present study (G*Power, Version 3.1.9.4) [25]. Thirty-four healthy, female physical education students (age: 20.03 ± 2.1 years, body mass: 60.9 ± 5.6 kg, body height: 164.1 ± 4.5 cm) participated and completed the study after being thoroughly informed, both orally and in written form, about the aims and the procedures of the study. The participants were in good health with no musculoskeletal injuries, did not receive nutritional supplements, and did not have any experience with systematic resistance or aerobic training for at least 2 years prior to the initiation of the present study. All the participants signed an informed consent clearly stating that they were free to withdraw from the study at any time point without stating the reason. All the procedures were according to the Declaration of Helsinki as revised in 2000 and were approved by the approved by the Ethics Committee of the School of Physical Education & Sports Science, the National and Kapodistrian University of Athens (number 1371/20-04-2022).

### 2.3. Procedures

#### 2.3.1. Concurrent Resistance and Aerobic Training

During the period of systematic training (T1 to T2), all the participants in the training group performed a concurrent resistance and aerobic training session (in exactly that order), 2 times per week, with at least 72 h between sessions. Each training session lasted ~1 h. Each session started with a 5 min warm-up on a bicycle at 50 Watts followed by 5 min of stretching for the lower extremities. Then, they performed 1 set of 12 repetitions at 40% of 1-RM and 1 set of 6 repetitions at 50–60% of 1-RM in the leg press machine. This was followed by 4 sets of 6 repetitions at 80–85% of 1-RM in leg press with 3 min rest between the sets. During the first week of training, the participants performed 3 sets of 6 repetitions at 60% 1-RM leg press to avoid any extensive muscle damage due to unaccustomed activity. The external load was increased by 2.5–5% each week [26,27]. Ten minutes after the completion of the resistance exercise, the participants performed 10 sets of 60 s duration on a bicycle ergometer at 100% of their maximal aerobic power with 50 revolutions per minute dictated by a metronome [5,28]. The passive recovery between the sets was 60 s. The cycling workload (watts) was increased by 2.5–5% in every training session [5,28]. During the first training week, the participants performed only 5 sets of 60 s duration on a bicycle ergometer. During the first training session of the second week, they performed 7 sets of 60 s duration, which increased to 8 sets of 60 s duration in the second training session. Thereafter, all the participants performed 10 sets of 60 s duration. The participants of the GC did not follow any type of systematic training throughout the study. The rate of perceived exertion [29] was evaluated after the end of the second training session of each week (RPE, CR10 scale: 0: very very light to 10: very very heavy).

#### 2.3.2. Reduced Training and Complete Cessation of Exercise

After the end of the initial 12-week training period (T1 to T2), the participants of the training group were assigned into 3 groups and continued for another 12 weeks (T2 to T4) as follows: 20 participants performed the same concurrent resistance and aerobic training either once per week (G7; n = 10) or once every 14 days (G14; n = 10), while the rest 7 participants followed a complete training secession (GD; n = 7). The G7 and G14 groups continued their training with the training intensities and volumes used at the last training session of the regular training period, which was maintained in both groups until the end of the reduced training frequency period (T4). After the end of the reduced training frequency period, all the participants followed a 12-week period of complete training cessation (T4 to T5).

#### 2.3.3. Evaluation of Body Composition

The participants reported at the laboratory during morning hours at a fasted state for the evaluation of their body composition through dual-energy x-ray absorptiometry (Prodigy Pro, General Electric, Madison, WI, USA). They were asked to abstain from strenuous physical activities for at least 24 h before the evaluation. All the measurements were analyzed using the Lunar encore v.18 software for the determination of lean body mass (LBM), lower extremities’ lean mass, fat mass, and bone mass density (BMD). The intraclass correlation coefficients (ICCs) for the body mass, fat mass, and LBM were 0.99 for all the variables.

#### 2.3.4. Performance Evaluations

Jumping Performance. The muscle power of the lower extremities was evaluated through a countermovement jumping test. The test was performed on a force platform (Applied Measurements Ltd., Co., Aldermaston UK, WP800 80 × 80 cm, sampling frequency 1 kHz). After a 10 min warm-up on a stationary bicycle at 50 Watts, the participants performed 3 CMJs with submaximal intensity and then 3 maximal CMJs with 2 min rest between each jump, with arms akimbo. All the efforts were recorded and analyzed (Kyowa sensor interface PCD-320A) in order to calculate jump height [((0.5 × flight time) 2 × 2-1) × 9.81] and maximum power [(body weight + Fmax) × 9.81 × flight time]. The signal was filtered using a secondary low-pass Butterworth filter with a cutoff frequency of 10 Hz. The best performance according to the jump power was used for further analysis. The ICCs for jump height and power were 0.87 and 0.91.

Leg press 1-RM. The evaluations of lower extremities’ maximum strength were performed in a 45° incline leg press machine as previously described [5,26]. Thirty minutes after the evaluation of the jumping performance, the participants performed 1 set of 10 repetitions with approximately 40% of the predicted 1-RM to warm up. Then, they performed 3 sets of 8, 4, and 2 repetitions with approximately 50–60%, 70–75%, and 80–85% of the predicted 1-RM, respectively. Thereafter, 3–6 sets of single repetitions were performed for the determination of their maximum strength. Three minutes of rest was allowed between the sets while two of the researchers were present for monitoring the technique of the exercise and to encourage the participants to perform their best. The rate of perceived exertion (CR10 scale: 0: very very light to 10: very very heavy) [29] was recorded after each set. The ICC of this evaluation is 0.98 in our laboratory [5,26].

Maximum aerobic power. Maximum aerobic power was evaluated on a bicycle ergometer (Monark 834E; Monark Exercise AB, Vansbro, Sweden) 2 days after the evaluations of CMJ and 1-RM performances. The test was based on the adjusted YMCA Cycle Ergometer Protocol [30] as it has been previously used in our laboratory [5,28]. Specifically, after 5 min of static and dynamic stretching for the lower extremities, the participants started to cycle at 25 Watts with 50 revolutions per minute. The increase in resistance in the second 3 min stage depended on the first-stage heart rate (HR): if HR was under 80 beats per minute (b/min) at the end of the first stage, the resistance in the second stage was increased 100 Watts, if HR fluctuated between 80 and 90 b/min the resistance was increased 75 Watts, if HR fluctuated between 90 and 100 b/min the resistance was increased 50 Watts, and if HR was over 100 b/min the resistance was increased 25 Watts. From the third 3 min stage and on, the resistance was increased by 25 Watts until exhaustion (when each participant could no longer maintain the 50 revolutions per minute). Heart rate was monitored and recorded throughout the test (Polar A300, Polar Electro, Kampele, Finland). The rate of perceived respiratory and leg exertion was evaluated at the end of each 3 min stage through a CR10 scale (0: very very light to 10: very very heavy) [29,30]. After the end of the test, the participants continued pedaling for 3–5 min at 25 Watts. The maximum aerobic power and maximum heart rate were defined as those achieved during the last completed stage [5,28]. The ICC for this test was 0.91 [5,28].

#### 2.3.5. Ultrasonography

The participants were placed for 10 min at the supine position before the initiation of ultrasonography. B-mode axial-plane ultrasound images (LOGIQ S9, General Electric, Boston, MA, USA) were taken with an ML6-15 MHz linear-array probe with the extended field of view mode. For the quadriceps CSA imaging, a line from the center of the patella to the medial aspect of the anterior superior iliac spine was marked and then an axial perpendicular line was drawn at 40% of this distance (proximal to the knee). Based on this marked line, the probe was moved transversely across the thigh taking a continuous single view which pictured the CSA of the whole quadriceps with its four heads separately [5,31]. The CSA of the whole quadriceps femoris and each separate section (vastus lateralis-VL, rectus femoris-RF, vastus intermedius-VI, and vastus medialis-VM) was analyzed using an appropriate image analysis software (ImageJ Version 1.54d National Institutes of Health). The ICC for the whole quadriceps femoris CSA, VL, RF, VI, and VM has been calculated to be 0.97, 0.96, 0.94, 0.95, and 0.87, respectively.

For the measurement of the vastus lateralis architecture, B-mode ultrasound images were taken at 50% of the distance from the central palpable point of the greater trochanter to the lateral condyle of the femur [32,33] using the extended field of view mode. The transducer was placed longitudinally on the femur, oriented parallel to the muscle fascicles and perpendicular to the skin. The images were analyzed for muscle thickness, fascicle angle, and fascicle length with the image analysis software (ImageJ Version 1.54d National Institutes of Health). Muscle thickness was defined as the distance between the superficial and deep aponeurosis, fascicle angle as the angle of insertion of muscle fascicles onto the deep aponeurosis, and fascicle length as the fascicular path between the insertions of the fascicle onto the upper and deeper aponeurosis. The ICC for muscle thickness, fascicle length, and fascicle angle in our laboratory has been calculated to be 0.97, 0.84, and 0.88, respectively. 

### 2.4. Statistical Analyses 

All the data are presented as means and standard deviation (±SD). The Shapiro/Wilks test was used to check the normality of the data. Νo violation was found. Student’s T-tests were used to detect percentage change differences pre- to post systematic training for the initial training period (T1 to T2). A two-way repeated analysis of variance (ANOVA) was used to test the interaction between training intervention and time (T1 to T5). Bonferroni post hoc analysis was used when it was necessary. Effect sizes were calculated (Hedges’ g). The level of statistical significance was set at *p* ≤ 0.05. The statistical analyses were performed with SPSS version SPSS 25.0 (SPSS Inc., Chicago, IL, USA).

## 3. Results

### 3.1. Systematic Concurrent Training (T1 to T2)

**Figure 2:**
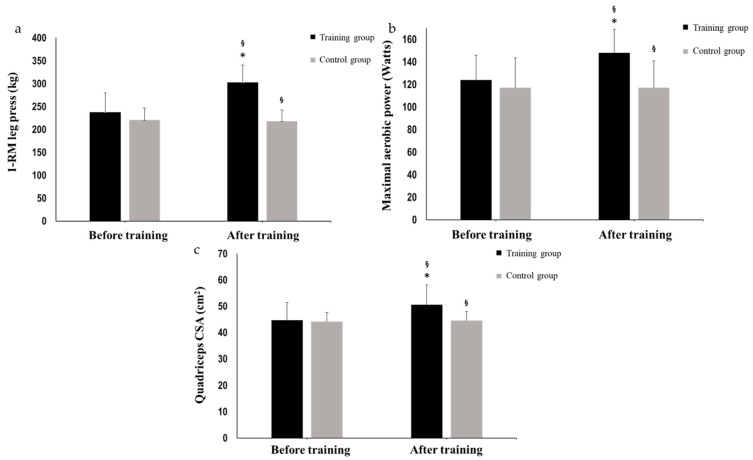
Leg press 1-RM (**a**), maximal aerobic power (**b**), and quadriceps CSA (**c**) at the start of the training period (T1) and after 12 weeks of systematic training (T2) in the training and the control group. (*) denotes the significant difference after training in each group separately and (§) denotes the differences between the groups at the marked time points. *p* < 0.05.

Physical performance. Leg press 1-RM, maximal aerobic power, and HR at 100 and 125 Watts during the aerobic test was significantly increased from T1 to T2 in the training group (28.7 ± 10.7%, *p* < 0.001, Hedges’ g = 1.630; 20.4 ± 10.3%, *p* < 0.001, Hedges’ g = 1.077; 10.1 ± 6.9%, *p* < 0.001, Hedges’ g = 1.084; and 10 ± 4.1%, *p* < 0.001, Hedges’ g = 1.685; respectively, Table 1; Figure 2). The lean body mass of lower extremities was increased significantly from T1 to T2 in the training group (5.0 ± 2.7%, *p* < 0.001, Hedges’ g = 0.339; Table 1). In the above parameters, significant differences between the groups were found at T2 (*p* < 0.05; Table 1; Figure 1). There was no significant change for the training group in CMJ muscle power and jump height from T1 to T2 (*p* > 0.05; Table 1). Fat mass, total lean body mass, and bone mineral density remained unchanged for both groups between T1 and T2 (*p* > 0.05; Table 1). There was no significant change for the control group in any of the above parameters.

Quadriceps muscle CSA and architecture. The CSA of the whole quadriceps muscle (13.1 ± 4.7%, *p* < 0.001, Hedges’ g = 0.809; Table 1; Figure 1), as well as for each of the quadriceps heads (VL: 3.7 ± 7.5%, *p* < 0.001, Hedges’ g = 1.000; RF: 16.5 ± 9.9%, *p* < 0.001, Hedges’ g = 0.694; VI: 11.2 ± 6.7%, *p* < 0.001, Hedges’ g = 0.589; VM: 14.5 ± 9%, *p* < 0.001, Hedges’ g = 0.702; Table 1), were significantly increased from T1 to T2 only in the training group. No significant changes were observed in any group after the initial training period for vastus lateralis fascicle length (Table 1). Fascicle angle and vastus lateralis thickness were significantly increased from T1 to T2 only in the training group (17.1 ± 12.2%, *p* < 0.001, Hedges’ g = 1.085; 10.7 ± 3.3%, *p* < 0.001, Hedges’ g = 1.000; respectively, Table 1). 

### 3.2. Reduced Training Frequency and Detraining (T2 to T5)

**Figure 3:**
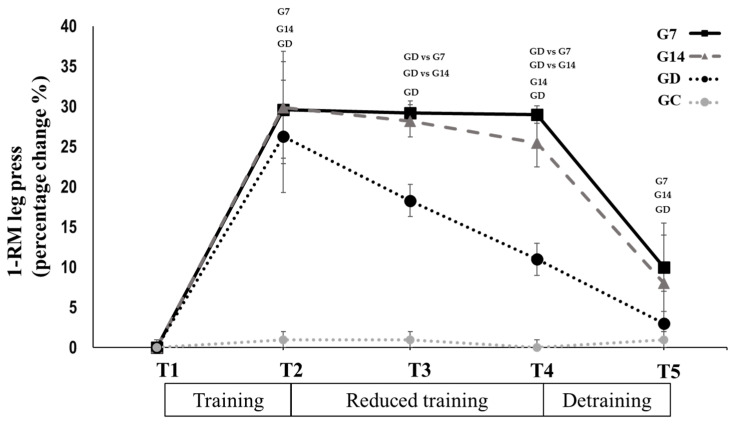
Leg press 1-RM percentage changes after 12 weeks of systematic training (T1 to T2), after 6 weeks (T2 to T3) and 12 weeks of reduced frequency training (T2 to T4), and after 12 weeks of detraining (T4 to T5) for the G7, G14, GD, and GC groups. Small letters denote statistically significant differences in the marked group separately (where G7 = 1 training session every 7 days, G14 = 1 training session every 14 days, and GD = exercise cessation) between time periods (T1 to T2, T3 to T4, and T4 to T5). When a comparison between groups (for example GD vs. G7) is presented, it refers to the significant differences between the denoted groups in the marked time points.

Physical performance: Leg press 1-RM in G7 (−0.4 ± 1%, *p* = 0.193, Hedges’ g = 0.036; Table 2) and G14 (−1.4 ± 2%, *p* = 0.056, Hedges’ g = 0.149; Table 2) remained unchanged from T2 to T3. In contrast, it changed significantly in GD (−9.1 ± 2.3%, *p* = 0.001, Hedges’ g = 1.048; Table 2). Leg press 1-RM (−0.5 ± 1.2%, *p* = 0.168, Hedges’ g = 0.040; Table 2) remained unchanged from T3 to T4 in G7. In contrast, it changed significantly in G14 (−2.9 ± 3.1%; *p* = 0.009, Hedges’ g = 0.342; Table 2) and GD (−6.8 ± 3.2%, *p* = 0.006, Hedges’ g = 0.688; Table 2). The percentage changes in 1-RM were not significantly different between G7 and G14, while the greatest changes were observed in GD (*p* < 0.05). During the period between T4 and T5, leg press 1-RM decreased in G7 (−17.4 ± 5.9%, *p* = 0.001, Hedges’ g = 1.286; Table 2), G14 (−16.5 ± 6.8%, *p* = 0.025, Hedges’ g = 1.418; Table 2), and GD (−9.3 ± 4.6%, *p* = 0.002, Hedges’ g = 0.843; Table 2) with all the groups reaching the pretraining values (Figure 3). There was no significant change for the control group in any time period.

**Figure 4:**
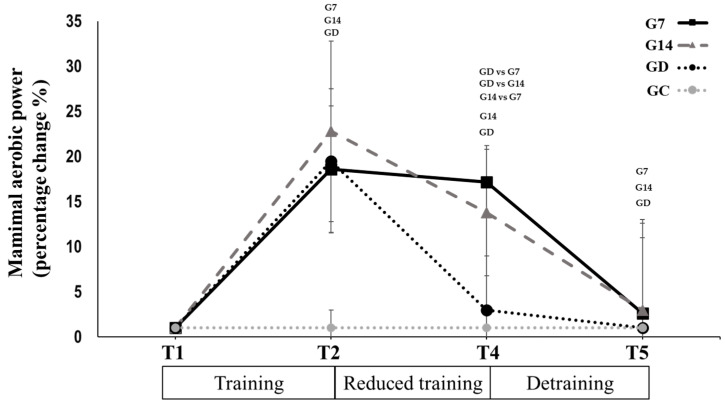
Maximal aerobic power percentage change after 12 weeks of systematic training (T1 to T2), 12 weeks of reduced frequency training (T2 to T4), and after 12 weeks of detraining (T4 to T5) for G7, G14, GD, and GC groups. Small letters denote statistically significant differences in the marked group separately (where G7 = 1 training session every 7 days, G14 = 1 training session every 14 days, and GD = exercise cessation) between time periods (T1 to T2, T2 to T4, and T4 to T5). When a comparison between groups (for example GD vs. G7) is presented, it refers to the significant differences between the denoted groups in the marked time points.

Cardiovascular performance: Maximal aerobic power (−1.4 ± 4.5%, *p* = 0.343, Hedges’ g = 0.096; Table 3) and heart rate at 100 Watts (+3.5 ± 5.1%, *p* = 0.064, Hedges’ g = 0.362; Table 3) and 125 Watts (+1.5 ± 4.2%, *p* = 0.320, Hedges’ g = 0.178; Table 3) remained unchanged from T2 to T4 in G7. In contrast, they changed significantly in G14 (−9.0 ± 7.8%, Hedges’ g = 1.024; +12.2 ± 15.8%, Hedges’ g = 1.085; and +11.2 ± 8.6%, Hedges’ g = 1.016, respectively, *p* < 0.030; Table 3), while they returned to pretraining values in GD (−15.8 ± 7.3%, Hedges’ g = 1.231; +9.7 ± 2.9%, Hedges’ g = 1.606; and +7.3 ± 7.2%, Hedges’ g = 1.288, respectively, *p* < 0.049; Table 3). In all the cases, the percentage changes in the above parameters significantly differed between groups, with G7 demonstrating to lowest changes, while the greatest changes were observed in GD (*p* < 0.05). During the period between T4 and T5, maximal aerobic power decreased in G7 (−14.5 ± 15.3%; *p* = 0.011, Hedges’ g = 0.696; Table 3) and G14 (−10.3 ± 11.9%; *p* = 0.024, Hedges’ g = 0.978; Table 3), with both groups reaching the pretraining values. During this period, heart rate at 100 Watts and 125 Watts increased significantly in G7 (+8.1 ± 3.1%, Hedges’ g = 0.529 and +4.8 ± 4.1%, Hedges’ g = 0.493, respectively, *p* < 0.004; Table 3) reaching the pretraining values while they did not change any further in G14 and GD (*p* > 0.05; Table 3; Figure 4). There was no significant change for the control group in any time period.

Jumping performance: No significant changes in jumping performances were observed in any group for the periods between T2 and T5 (*p* > 0.05). Training intensity and volume remained unchanged throughout the whole reduced training frequency period, although the participants reported higher rating of perceived exertion (RPE) values during the period of reduced training (RPE at T2 8.2 ± 0.8 vs. RPE at T4 9.1 ± 0.6, *p* < 0.05).

Body Composition: The lean mass of the lower extremities (−0.3 ± 1.8%, *p* = 0.502, Hedges’ g = 0.052; Table 3) remained unchanged from T2 to T4 in G7. In contrast, it decreased significantly in G14 and GD (−1.76 ± 2%, *p* = 0.018, Hedges’ g = 0.166 and −1.85 ± 3.6%, *p* = 0.011, Hedges’ g = 0.399, respectively; Table 3), with GD reaching the pretraining values. The percentage changes in the lean mass of the lower extremities were not significantly different between the groups. During the period between T4 and T5, the above parameter decreased in G7 (−3.05 ± 3.9%, *p* = 0.044, Hedges’ g = 0.210; Table 3) and G14 (−2.29 ± 3.3%, *p* = 0.040, Hedges’ g = 0.181; Table 3) with both groups reaching the pretraining values while there was no change in GD (*p* = 0.081; Table 3). Fat mass, lean body mass, and bone mineral density remained unchanged for all the groups between T2-T5 (*p* > 0.05; Table 3). There were no significant changes in the control group in any time period.

**Figure 5:**
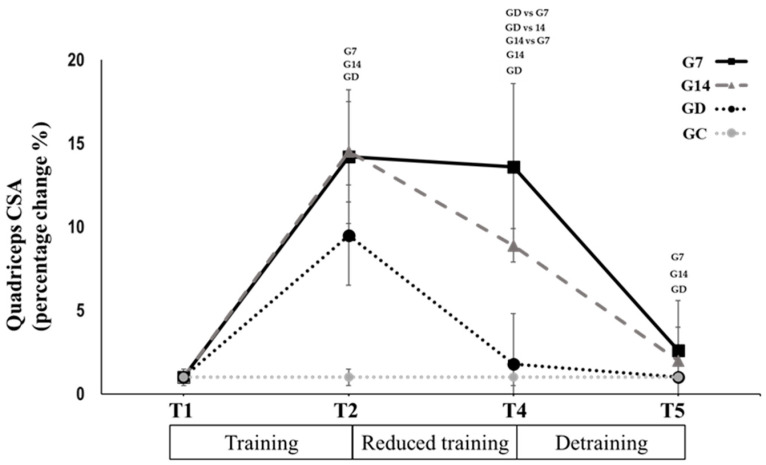
Quadriceps CSA percentage change after 12 weeks of systematic training (T1 to T2), 12 weeks of reduced frequency training (T2 to T4), and after 12 weeks of detraining (T4 to T5) for G7, G14, GD, and GC groups. Small letters denote statistically significant differences in the marked group separately (where G7 = 1 training session every 7 days, G14 = 1 training session every 14 days, and GD = exercise cessation) between time periods (T1 to T2, T2 to T4, and T4 to T5). When a comparison between groups (for example GD vs. G7) is presented, it refers to the significant differences between the denoted groups in the marked time points.

Quadriceps muscle CSA and Vastus Lateralis architectural characteristics: The CSA of the whole quadriceps muscle and of each of the quadriceps heads remained unchanged from T2 to T4 in the G7 (−0.6 ± 0.6%, *p* = 0.376; *p* < 0.005, Hedges’ g = 0.032–0.060; Table 3). In contrast, they decreased significantly in G14 (−5.9 ± 1.8%, *p* = 0.001; *p* < 0.01, Hedges’ g = 0.396–0.469; Table 3), while they returned to the pretraining values in GD (−7.6 ± 3.4%; *p* = 0.001; *p* < 0.01, Hedges’ g = 0.548–0.669; Table 3). In all the cases, the percentage changes in the above parameters significantly differed between groups, with G7 demonstrating to lowest changes, while the greatest changes were observed in GD (*p* < 0.05; Figure 5). During the period between T4 and T5, the above parameters decreased in G7 (−11.5 ± 3.2%; *p* = 0.001; *p* < 0.01, Hedges’ g = 0.530–0.680; Table 3) and in G14 (−6.7 ± 2.2%; *p* = 0.001; *p* < 0.01, Hedges’ g = 0.427–0.583; Table 3), with both groups reaching the pretraining values, while they did not change any further in GD (*p* = 0.156; Hedges’ g = 0.045–0.077; Table 3). There was no significant change for the control group in any time period. 

Vastus lateralis thickness (0.5 ± 0.9%; *p* = 0.111; Hedges’ g = 0.010; Table 3) and fascicle angle (−1.5 ± 3.3%, *p* = 0.179; Hedges’ g = 0.166; Table 3), remained unchanged from T2 to T4 in G7. In contrast, they changed significantly in G14 (−5.2 ± 2.3%, *p* = 0.001, Hedges’ g = 0.500 and −7.51 ± 4%, *p* = 0.013, Hedges’ g = 0.500, respectively; Table 3), while they returned to the pretraining values in GD (−8.4 ± 2.6%, *p* = 0.001, Hedges’ g = 1.264 and −8.7 ± 5.1%, *p* = 0.011, Hedges’ g = 1.075, respectively; Table 3). In all the cases, the percentage changes in the above parameters significantly differed between groups, with G7 demonstrating to lowest changes, while the greatest changes were observed in GD (*p* < 0.05). During the period between T4 and T5, the above parameters decreased in G7 (−10.5 ± 2.6%; *p* = 0.001, Hedges’ g = 1 and −13.3 ± 8.2%; *p* = 0.001, Hedges’ g = 1.154, respectively; Table 3) and in G14 (−4.5 ± 2.9%; *p* = 0.001, Hedges’ g = 0.500 and −7.79 ± 8.4%; *p* = 0.001, Hedges’ g = 0.558, respectively; Table 3), reaching the pretraining values, while they did not change any further in GD (*p* = 0.092 and *p* = 0.084, Hedges’ g = 0.095, respectively; Table 3). Fascicle length did not change significantly after either time period in any group (*p* > 0.05; Table 3). There was no significant change for the control group in any time period.

## 4. Discussion

The present study aimed to investigate whether concurrent training once every 7 or 14 days during a period of forced reduction in the training frequency would be adequate to at least partly preserve the training-induced adaptations after a period of systematic concurrent training. To our knowledge, this is the first study evaluating the effect of two different reduced concurrent training frequencies on muscle strength and morphology in young females. According to the results of the present study, it seems that muscle strength is completely preserved even if a training stimulus is provided every 14 weeks, at least for the first 6 weeks. However, after a 12-week period of forced reduced training frequency, it seems that muscle strength, muscle mass, and aerobic power could only be preserved at the same levels as those at the end of a systematic training period if training stimulus is provided every 7 days, and not if it is provided every 14 days. Yet, even if the training stimulus is provided every 14 days, most of the resistance training-induced improvements are preserved. High-intensity resistance training once per week is known to preserve muscle strength for at least 12 weeks [19,20,23,34,35]. Here, we show that concurrent resistance and aerobic training once per week may fully preserve the training-induced adaptations in muscle strength/morphology and cardiopulmonary endurance; however, even a training stimulus every 14 days is capable of preserving >90% of the training-induced adaptations in the above parameters. In contrast, in the detraining group, almost all the training-induced adaptations were lost after 12 weeks of complete exercise cessation. The same results were found in both the G7 and G14 groups during their detraining period, independently of the training group during the reduced training frequency period. Actually, in all the cases, detraining resulted in a complete loss of all the physiological and performance adaptations except muscle strength that remained slightly above baseline levels. This is in concert with previous studies showing that 12 weeks of exercise cessation is enough for a complete loss of muscle strength [36,37]. 

Likewise, one concurrent training session every 14 days for 12 weeks preserved approximately 94% of the quadriceps muscle CSA, VL thickness, and fascicle angle training adaptations. The maintenance of muscle mass is a critical performance and health-related element [2] and the current results have practical applications for training enthusiasts who have limited time and/or opportunities for systematic training, at least for a brief time period. As expected, training once every week for 12 weeks preserved all of the training adaptations in quadriceps muscle CSA, VL thickness, and fascicle angle. This is in concert with previous studies showing similar results after 8–20 weeks of reduced training frequency, from 3/week to 2/week or from 2/week to 1/week [19,20,23]. In contrast, all the muscle mass gains were lost after 12 weeks of complete exercise cessation for the detraining group of the current study, an observation which was expected based on the results of previous studies [14,36,38]. 

Maximal aerobic power was also partly preserved (approximately 91%) with concurrent training every 14 days. Thus, individuals with time restrictions for certain time periods may maintain a large part of their aerobic adaptations by training just once every 2 weeks. As expected, concurrent training once every week was an adequate stimulus for the preservation of maximal aerobic power as well as for submaximal heart rate adaptations. Previous studies have shown that the intensity of aerobic training has a significant role in preserving the adaptations in aerobic power during a period of reduced training frequency [39,40]. In contrast, all the aerobic gains were retracted after 12 weeks of complete exercise cessation in the detraining group, an observation which is in line with previous reports in young women [41]. However, we do not know how the biological factors that affect aerobic capacity changed at different time periods. Moreover, we do not know what the magnitude of the adaptations and the loss of the adaptations in each of these factors were and how much the changes in each of these factors affected the results of this research.

The preservation of the training-induced adaptations during a short-term period of reduced training frequency seems to depend on the training intensity and volume. Indeed, it has been reported that if the training intensity and volume are kept at the same levels as those performed during the systematic training period, the majority of the training-induced adaptations will be maintained, at least if the training stimulus is provided once every week [17,20]. According to the results of the present study, it seems that even if the training stimulus is provided once every 14 days with the same training intensity and volume as that which was used during the period of systematic training, most of the training-induced improvements are preserved up to 90–95%. Intensity is a key factor for increasing muscle strength. This is probably because strength is largely dependent not only on muscle mass but also on other factors such as neuromuscular factors (e.g., fast twitch fiber recruitment, etc.) that benefit more from explosive and/or high load actions [42]. Moreover, training volume seems to be one of the most important factors for muscle hypertrophy, with high training volumes leading to significantly greater adaptations in muscle mass independently of the intensity of the external load as long as individuals are trained to or at least close to muscle failure [43]. For example, it has been reported that both low loads with high repetitions (20–25 repetitions with 30–50% RM) and high loads with few repetitions (8–12 repetitions with 75–90% RM) resulted in the same improvements in muscle mass if both protocols have the same total training workload and are performed until muscle failure [42].

Concurrent resistance and high-intensity aerobic training induce muscle strength, muscle mass, and aerobic adaptations [5,28]. The current study is along the lines of these previous data. After 12 weeks of resistance and aerobic training, significant increases were found in muscle strength, muscle mass, and aerobic power. The addition of high-intensity cycling to resistance exercise prompted significant cardiovascular adaptations leading to improved aerobic adaptations, as also found in previous studies showing that resistance exercise does not inhibit aerobic power adaptations expressed either as VO2max, maximal aerobic power, or time to exhaustion [43,44,45,46]. Also, high-intensity aerobic training (bicycling) does not inhibit muscle strength and muscle mass adaptations; on the contrary, it is a potent stimulus for the muscle hypertrophy of the quadriceps [5,28]. The muscle power of the lower extremities was not altered after 12 weeks of systematic concurrent training despite muscle strength increases. The addition of aerobic training after resistance training seems to inhibit the adaptations in jumping performance [28,47,48,49,50]. Other biological adaptations, beyond increases in muscle strength and muscle hypertrophy, may be responsible for the lack of change in jumping performance with this type of concurrent training. 

The present study has some significant limitations. Unfortunately, it was not feasible to evaluate the neuromuscular adaptations through electromyography during the training and detraining periods. Furthermore, no muscle biopsies were taken in the present study in an effort to evaluate the training-/detraining-induced adaptation of muscle fiber composition and size or to understand the physiological/biochemical/molecular background of the observed results. Furthermore, unfortunately, it was not possible in this study to examine the physiological background changes in aerobic capacity.

## 5. Conclusions

The results of the present study suggest that muscle strength and the mass of the lower extremities as well as aerobic power are well preserved with just three training sessions in 6 weeks (once every 14 days) after a period of systematic concurrent training. If this bimonthly training frequency is continued for 6 more weeks, both muscle strength/mass and aerobic power decrease significantly, yet most of the resistance training-induced improvements are preserved. Therefore, performing one training session every 14 days for 3 months after a period of systematic concurrent resistance and aerobic training may preserve up to 90–95% of the muscle strength, muscle mass, and aerobic power adaptations achieved during the systematic training period if the training intensity and volume are sufficient. Thus, when practitioners are forced to reduce their exercise frequency, even one training session every 14 days can be beneficial in preserving the exercise-induced benefits in performance and health. However, if an individual performs one training session per week, it seems that all of their training-induced adaptations on muscle mass, morphology, strength, and aerobic performance could be kept at the same point as those during the end of a systematic training period, at least for the first 12 weeks of a forced reduced training frequency period. The results of the present study provide practical applications for individuals seeking to maintain their training-induced adaptation on muscular strength and hypertrophy as well as on cardiovascular endurance, but experiencing difficulties following a frequent training routine, for at least some period. Probably, even if a low training frequency, equal to one training session per 14 days, is followed, it would make it easier for these individuals to rejoin systematic training after some weeks of reduced training frequency with a minimal loss of training-induced adaptations.

## Figures and Tables

**Table 1:** Body composition, vastus lateralis architecture, quadriceps cross-sectional area, and performance before and after the initial 12-week concurrent training period (T1 to T2).

		Training Group (N = 27)		Control Group (Ν = 7)	
		Before	After	Before	After
Fat mass (kg)		17.6 ± 4.2	17.9 ± 4.1	17.8 ± 2.8	18 ± 1.8
Total LBM (kg)		40.7 ± 3.8	40.9 ± 4.2	38.4 ± 2.2	37.7 ± 2.4
LBM of lower extremities (kg)		13.8 ± 1.7	14.5 ± 1.8 *^,#^	13.3 ± 1.5	12.7 ± 1.4 ^#^
Bone mass density		1.208 ± 0.07	1.212 ± 0.09	1.219 ± 0.08	1.220 ± 0.06
VL fascicle length (cm)		6.6 ± 0.5	6.7 ± 0.7	6.5 ± 0.4	6.5 ± 0.4
VL fascicle angle (°)		16.1 ± 2.4	18.6 ± 2.2 *^,#^	16.1 ± 4.0	16.9 ± 4.1 ^#^
VL thickness (cm)		1.8 ± 0.2	2.0 ± 0.2 *^,#^	1.8 ± 1.0	1.8 ± 1.1 ^#^
CSA Quad (cm^2^)		44.7 ± 6.7	50.5 ± 7.6 *^,#^	44.2 ± 3.4	44.5 ± 3.5 ^#^
CSA VL (cm^2^)		14.2 ± 2.1	16.3 ± 2.1 *^,#^	14.1 ± 2.0	14.3 ± 2.1 ^#^
CSA RF (cm^2^)		4.7 ± 1.1	5.5 ± 1.2 *^,#^	3.9 ± 0.6	3.8 ± 0.6 ^#^
CSA VI (cm^2^)		15.9 ± 2.9	17.7 ± 3.2 *^,#^	16.3 ± 2.1	16.6 ± 2.1 ^#^
CSA VM (cm^2^)		9.5 ± 1.9	10.8 ± 1.8 *^,#^	9.6 ± 1.6	9.6 ± 1.8 ^#^
CMJ power (W)		1669 ± 453	1681 ± 443	1186 ± 300	1138 ± 325
CMJ height (cm)		19.4 ± 3.7	19.6 ± 3.8	17.3 ± 1.8	16.7 ± 1.7
Maximal aerobic power (W)		124.5 ± 22.4	148.0 ± 21.2 *^,#^	117.2 ± 12.2	117.4 ± 12.7 ^#^
Heart rate	100 W	153.1 ± 15.3	137.2 ± 14.1 *^,#^	151.5 ± 8.2	155.4 ± 9.3 ^#^
	125 W	166.5 ± 11.3	148.2 ± 10.4 *^,#^	169.9 ± 11.2	173.5 ± 7.7 ^#^
1-RM leg press (Kg)		237.9 ± 41.9	302.9 ± 37.7 *^,#^	220.7 ± 26.3	218.5 ± 24.2 ^#^

The values are presented as mean ± standard deviation. Significant differences between the T1 and T2 periods for each group are denoted by (*), while (^#^) denotes the significant differences between the groups at the marked time points. *p* < 0.05. LBM = lean body mass, VL = vastus lateralis, VI = vastus intermedius, VM = vastus medialis, RF = rectus femoris, CSA = cross-sectional area, and CMJ = countermovement jump.

**Table 2:** 1-RM leg press in G7, G14, and GD after 6 and 12 weeks of reduced frequency training (T2 to T4) and after 12 weeks of detraining (T4 to T5).

**1-RM leg press (Kg** **)**	**G7 (N = 10)**			
	**T2**	**T3**	**T4**	**T5**
	314 ± 41.4	312.5 ± 41.1	310.5 ± 40	257 ± 43.1 *^,‡^
	**G14 (N = 10)**			
	308 ± 35.7	303 ± 30.9	293 ± 27.4 *^,±^	246 ± 38 *^,‡^
	**GD (N = 7)**			
	280.4 ± 26.4	254.2 ± 23.5 *^,G7,G14^	237.1 ± 26.1 *^,±,G7,G14^	215 ± 26.3 *^,‡^

Here (*) denotes the significant difference between T3, T4, and T5 with T2 within each group. (^±^) denotes the significant differences in the T4 period in relation to the T3 period for each group separately. (^‡^) denotes the significant differences in the T5 period in relation to the T4 period for each group separately. (^G7^) denotes the significant differences in the rate of decline in 1-RM of the T4 period in group GD compared with group G7. (^G14^) denotes the significant differences in the rate of decline of the T4 period in group GD compared with group G14.

**Table 3:** Fat mass, total lean body mass, lean body mass of the lower extremities, bone mineral density, vastus lateralis architecture, quadriceps cross-sectional area, and performance parameters in G7, G14, and GD after 12 weeks of reduced frequency training (T2 to T4) and after 12 weeks of detraining (T4 to T5).

		G7 (N = 10)			G14 (N = 10)			GD (N = 7)		
		T2	T4	T5	T2	T4	T5	T2	T4	T5
Fat mass (kg)		15.5 ± 2.7	14.8 ± 2.9	15.6 ± 2.8	19.5 ± 4.4	19.8 ± 5.1	20.5 ± 4.7	20.1 ± 3.5	19.8 ± 4.4	20.7 ± 4.7
LBM (Kg)		40.9 ± 3.9	40.9 ± 3.9	40.1 ± 3.8	41.6 ± 4.3	41.5 ± 4	41.3 ± 4.6	39.5 ± 4.8	39.3 ± 4.6	38.5 ± 4.5
LBM LE (Kg)		14.4 ± 1.9	14.3 ± 1.9	13.9 ± 1.9 *^,±^	14.9 ± 1.9	14.6 ± 1.7 *	14.3 ± 1.6 *^,±^	14.1 ± 1.6	13.5 ± 1.4 *	13.5 ± 1.5 *
BMD		1.227 ± 0.1	1.230 ± 0.1	1.228 ± 0.1	1.224 ± 0.1	1.226 ± 0.1	1.222 ± 0.1	1.174 ± 0.7	1.175 ± 0.7	1.173 ± 0.7
VL FL (cm)		6.4 ± 0.9	6.6 ± 0.9	6.4 ± 0.7	6.7 ± 0.6	6.8 ± 0.4	6.8 ± 0.5	6.9 ± 0.3	6.8 ± 0.4	6.8 ± 0.4
VL FA (°)		18.4 ± 1.9	18.1 ± 1.7	15.7 ± 2.4 *^,±^	19.1 ± 2.8	17.7 ± 2.7 *^,G7^	16.3 ± 3.2 *^,±^	18.4 ± 2.0	16.7 ± 1.0 *^,G7^	16.6 ± 1.1 *
VL T (cm)		2.0 ± 0.2	2.0 ± 0.2	1.8 ± 0.2 *^,±^	2.0 ± 0.2	1.9 ± 0.2 *^,G7^	1.8 ± 0.2 *^,±^	2.0 ± 0.1	1.8 ± 0.1 *^,G7^	1.8 ± 0.1
QCSA (cm^2^)		49.9 ± 8.8	49.6 ± 9.2	43.8 ± 7.8 *^,±^	53.8 ± 6.6	50.7 ± 6.6 *^,G7^	47.1 ± 5.7 *^,±^	46.6 ± 5.5	43.3 ± 6.5 *^,G7,G14^	42.8 ± 6.4 *
CSA VL (cm^2^)		16 ± 2.8	16 ± 2.9	14.1 ± 2.1 *^,±^	17.7 ± 2.5	16.7 ± 2.4 *^,G7^	15.4 ± 2 *^,±^	14.8 ± 1.5	13.7 ± 1.6 *^,G7^	13.6 ± 1.5 *
CSA RF (cm^2^)		5.3 ± 1.3	5.2 ± 1.4	4.4 ± 0.9 *^,±^	5.7 ± 0.9	5.2 ± 0.9 *^,G7^	4.8 ± 0.9 *^,±^	5.4 ± 1.7	4.9 ± 1.6 *^,G7^	4.9 ± 1.6 *
CSA VI (cm^2^)		17.2 ± 3.7	17.2 ± 3.7	15.5 ± 3.1 *^,±^	19.3 ± 2.8	18.3 ± 2.2 *^,G7^	17 ± 2.5 *^,±^	16.1 ± 2.1	15.4 ± 2.5 *^,G7^	15.3 ± 2.5 *
CSA VM (cm^2^)		11.1 ± 1.9	11 ± 1.9	9.7 ± 2.4 *^,±^	10.9 ± 2.1	10.1 ± 2 *^,G7^	9.7 ± 1.3 *	10.1 ± 0.7	8.8 ± 1.4 * ^G7 G14^	8.7 ± 1.4 *
CMJ power (W)		1892 ± 585	1863 ± 461	1768 ± 509	1675 ± 274	1654 ± 294	1600 ± 217	1388 ± 222	1358 ± 232	1337 ± 289
CMJ height (cm)		22.5 ± 4.1	22.9 ± 3.9	22.5 ± 4.2	18.0 ± 1.8	17.8 ± 2.4	18.0 ± 1.9	17.7 ± 3.1	17.9 ± 2.3	16.9 ± 1.6
Maximal AP (W)		147.5 ± 24.8	145.2 ± 22.9	125.3 ± 33.3 *^,±^	157.5 ± 16.8	142.5 ± 12.1 *^,G7^	127.5 ± 18.0 *^,±^	135.7 ± 15.0	114.2 ± 19.6 *^,G7,G14^	107.5 ± 26.7 *
HR	100 W	141.2 ± 13.2	146.1 ± 13.8	152.5 ± 10.1 *^,±^	128.9 ± 16.0	142.7 ± 8.2 *	148.1 ± 14.3 *	145.1 ± 9.1	159.8 ± 9.2 *	162.8 ± 10.3 *
	125 W	160.2 ± 13.3	162.5 ± 12.2	167.5 ± 7.5 *^,±^	148.4 ± 14.1	160.2 ± 8.2 *^,G7^	164.7 ± 6.8 *	156.3 ± 8.0	167.0 ± 8.6 *^,G7^	167.2 ± 4.0 *

Here (*) denotes the significant difference between T4, T5, and T2 for each parameter within each group. (^±^) denotes the significant differences in the T5 period in relation to the T4 period for each parameter within each group. Small letters denote statistically significant differences between the marked groups at each time point (where ^G7^ = 1 training session every 7 days, ^G14^ = 1 training session every 14 days, and GD = exercise cessation). LBM = lean body mass, LBM LE = lean body mass of lower extremities, BMD = bone mass density, VL FL = vastus lateralis fascicle length, VL FA = vastus lateralis fascicle angle, VL T = vastus lateralis thickness, QCSA = quadriceps cross-sectional area, VL = vastus lateralis, RF = rectus femoris, VI = vastus intermedius, VM = vastus medialis, CMJ = countermovement jump, AP = aerobic power, and HR = heart rate: Standardized Beta coefficient of linear regression analysis.

## Data Availability

The original contributions presented in the study are included in the article, further inquiries can be directed to the corresponding author.
